# COVID-19 Pandemic and the Operation of a Tertiary Hospital in Tianjin—The Impact of Internet Healthcare on Hospital Business Indicators

**DOI:** 10.3389/fpubh.2022.892773

**Published:** 2022-05-20

**Authors:** Jia Chen, Liu Jie, Xiao Su, Xin Zhang, Dandan Guo, Jiajing Wang, Meili Deng, Mingrui Cui, Meijian Gao, Hong Zhang, Yijun Song

**Affiliations:** ^1^Department of Out-Patient and Emergency, General Hospital of Tianjin Medical University, Tianjin, China; ^2^Department of Neurology, Tianjin Medical University General Hospital, Tianjin, China; ^3^Laboratory of Epidemiology, Tianjin Neurological Institute, Tianjin, China; ^4^College of Management, Tianjin Normal University, Tianjin, China; ^5^College of Management, Sehan University, Yeongam-gun, South Korea; ^6^Department of General Medicine, Tianjin Medical University General Hospital, Tianjin, China; ^7^Tianjin Neurological Institute, Key Laboratory of Post-neuroinjury Neuro-Repair and Regeneration in Central Nervous System, Ministry of Education and Tianjin City, Tianjin, China

**Keywords:** COVID-19, medical service, medical security, Internet medical, trend analysis

## Abstract

**Objective:**

To explore the impact of the COVID-19 pandemic on hospital business and the contribution of Internet healthcare to hospital operations during the epidemic by analyzing the degree of impact on major business indicators.

**Methods:**

The three-year period from 2019 to 2021 was compared and analyzed, and the main medical business indicators such as outpatient and emergency visits, inpatients, operations, patient improvement rate, cure rate and fatality rate in tertiary hospitals were compared and analyzed, and the impact of the epidemic on medical services and hospital operation was analyzed. degree and the impact of Internet medical development on medical service capacity.

**Results:**

During the outbreak of COVID-19, the number of hospital outpatient and emergency visits, inpatients, and operations decreased significantly; after the normalization of the epidemic, the main medical business indicators such as outpatient and emergency visits, inpatients, and operations gradually returned to pre-epidemic levels; patient improvement rate, the cure rate and mortality rate and other indicators did not change significantly. During the epidemic period, the number of visits to the Internet outpatient clinic has increased significantly, which has significantly improved the hospital's medical service capacity.

**Conclusion:**

With the normalization of epidemic prevention and control, the main business indicators of Tianjin tertiary hospitals have gradually recovered. The operation of Internet medical care during the epidemic has changed the management and operation mode of the hospital to a certain extent, improved the main business indicators of the hospital, and eased the pressure on the hospital's economic operation.

## Introduction

The COVID-19 pandemic is the largest infectious disease outbreak in recent history and a major challenge to global health (1). In addition to the impact on human health of the COVID-19 pandemic, the greater threat is its indirect impact on institutions and individuals on the access and delivery of essential health services. In the extremely short months since the outbreak began in late 2019 and early 2020, more than one-third of the world's population has been under lockdown and movement restrictions (2). As a result of these restrictions, many patients have been denied access to health care; healthcare workers and other health resources have been reassigned to COVID-19, resulting in the suspension of many outpatient and inpatient services.

There is evidence that the pandemic has affected all aspects of health care delivery (3). For example, Pakistan reported a 52.5% drop in the average daily total number of vaccines administered during the lockdown compared to baseline (4). 10 A prospective observational study from Nepal reported a 51.4% reduction in facility-based deliveries with a corresponding increase in maternal and infant mortality (5). A national study in China found that during the worst phase of the epidemic, total health care spending and utilization dropped by 37.8% and 40.8%, respectively (6). A report involving 190 countries noted that during the first 12 weeks of the COVID-19 outbreak, 2,367,050 surgeries were canceled each week (7). Since the outbreak of the pandemic, IoT, blockchain, robotics and drones have gained recognition in the fight against the COVID-19 pandemic (8–10). Many robots have been used to fight the Covid-19 outbreak to avoid human interaction, monitoring, delivering items, spray disinfection, etc (10). Additionally, existing advanced technologies such as blockchain, digital twins and artificial intelligence can be combined to provide better and more precise pandemic alerts against the COVID-19 outbreak (8). Risk assessment can also be performed on patients with underlying medical conditions, and if infected, prompt action can be taken before seeking medical attention (11). In addition, internet medicine is considered an ideal tool to deal with this emergency. Globally, Internet telemedicine has been gradually implemented for about a decade. However, the lack of relevant laws and regulations, the limited economic investment of hospitals in technical resources, and the reluctance of some medical providers and patients to adopt telemedicine have made the development of Internet medical services slow (12–14). The COVID-19 pandemic has promoted and accelerated the development of Internet medical care (15).

In this study, we aimed to determine the impact of the COVID-19 pandemic on health by observing medical quality and safety indicators such as the number of outpatients, inpatients, operations, and patient cure rates, improvement rates, and case fatality rates in a tertiary hospital in Tianjin. The impact of critical functions of the system. To assess the impact of the COVID-19 pandemic on the continuum of health services in Tianjin, in particular to assess trends in health service utilization before and during the COVID-19 outbreak, and to explore the impact of internet healthcare on hospital management and operating models during the outbreak.

## Methods

### Data Collection

This study retrospectively analyzed the medical data of Tianjin Medical University General Hospital from January 2019 to December 2021. Combine the previous researches (5, 16, 17), medical service capacity indicators including the number of monthly admissions, the number of operations, the number of outpatient and emergency medical services; medical service safety and quality indicators including the cure rate, the fatality rate, and improvement rate. All data in this study come from the hospital information management department (linked to the medical insurance system), all patients have unique codes, and detailed medical records are also available for patients' admission routes and discharge information. All the collected data are used to analyze in this study. According to the time period, it is divided into T1 (January 2019 to November 2019), T2 section (December 2019 to April 2020), T3 section (May 2020 to December 2021). According to the outbreak time, the time period is divided into S1 (January 2019 to February 2020) before the outbreak and when the outbreak occurs, and S2 (March 2020 to December 2021) when the epidemic is normalized.

### Statistical Methods

Joinpoint performed trend analysis to calculate the change trend of different medical data. Joinpoint regression model was set the log linear model (ln y = xb), and choose unrelated model to fit. The grid search method modeling method was applied in this study. *P* < 0.05 was considered statistically significant. SPSS 25.0 and Joinpoint 4.9.0.0 were used for statistical analysis.

## Results

From January 2019 to December 2021, there were a total of 268,601 hospitalized patients, including 158,504 surgeries and 5,807 emergency hospitalizations. From June 2020 to December 2021, there were 742,514 Internet visits.

### Medical Service Capacity

**Figure 1:**
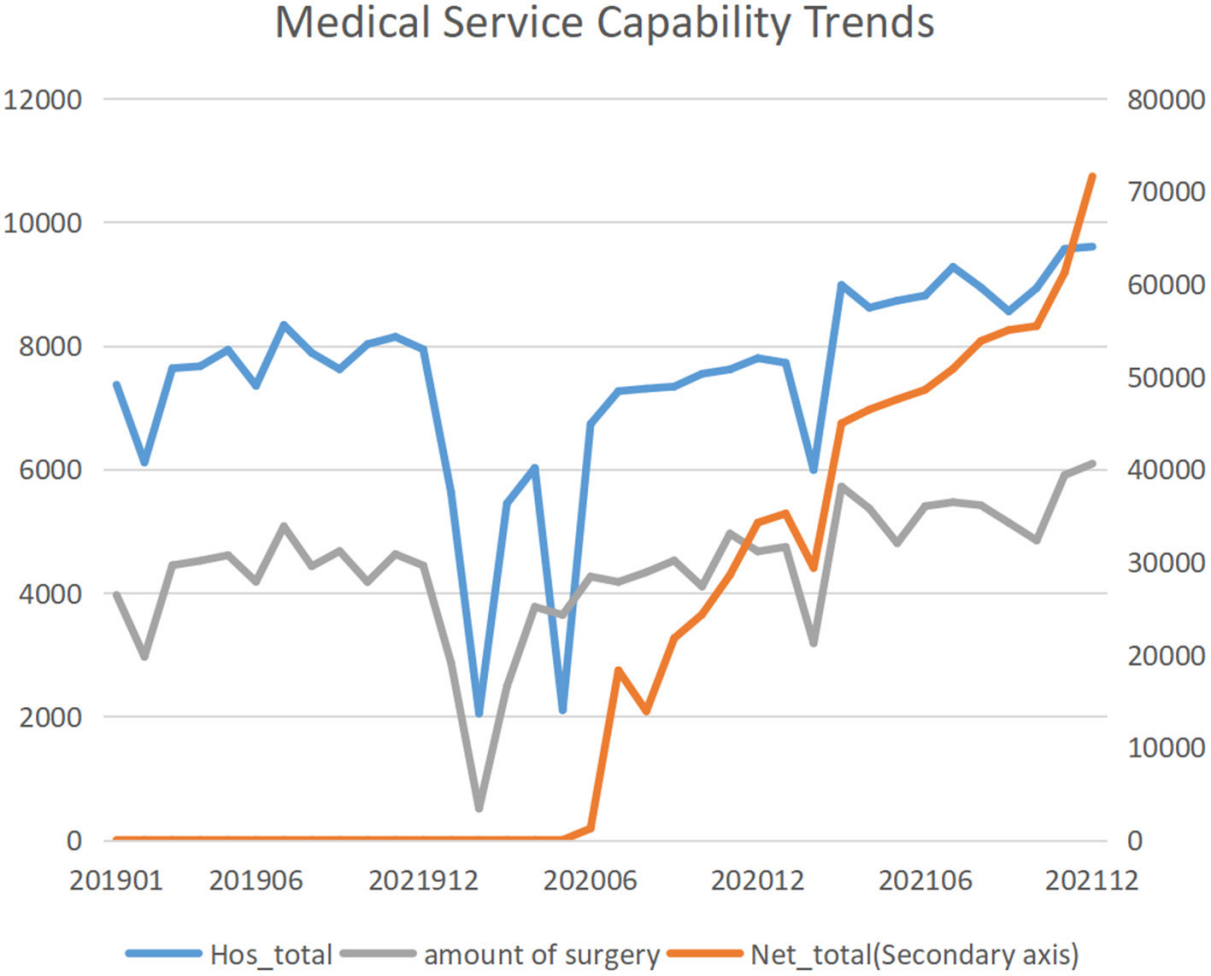
Medical service capability trends.

In this study, from January 2019 to December 2021, the number of hospitalizations showed a trend of first decreasing and then increasing, with the inflection point in February 2020. The number of hospitalizations in the S1 segment before and at the time of the outbreak decreased significantly (MPC: −3.5; 95%CI: −7.3, 0.4; *P* = 0.076), and the number of hospitalizations in the normalized S2 segment increased significantly (MPC: 3.6; 95%CI: 1.2, 6.0; *P* = 0.005). The trend of emergency hospital admissions and surgeries was similar to that of hospital admissions. The number of surgeries experienced a significant decrease at the time of the outbreak (MPC: −3.4; 95%CI: −6.4, −0.3; *P* = 0.032), and showed a significant trend of increasing after the epidemic normalized, with a monthly change percentage of 3.8% (95%CI: 2.2, 5.4; *P* < 0.001). Emergency hospital admissions also experienced a significant decrease during the outbreak, with a monthly percentage change of −2.9% (95% CI: −4.0, −1.7; *P* < 0.001). After the epidemic normalized, emergency admissions increased significantly, with a monthly percentage change was 2.3% (95% CI: 1.2, 3.3; *P* < 0.001). Since the opening of Internet clinics in June 2020, the number of Internet clinic visits has increased significantly, with a monthly percentage change of 13.0% (95% CI: 7.2, 19.1; *P* < 0.001) (Table 1; Figure 1).

**Table 1:** Trend analysis of medical service capability.

**Characteristics**	**MPC**	**95% CI**	***P***
**Hospitalization**			
S1	−3.5	(−7.3, 0.4)	0.076
S2	3.6	(1.2, 6.0)	0.005
**Number of operations**			
S1	−3.4	(−6.4, −0.3)	0.032
S2	3.8	(2.2, 5.4)	<0.001
**Emergency hospital admissions**			
S1	−2.9	(−4.0, −1.7)	<0.001
S2	2.3	(1.2, 3.3)	<0.001

*S1, The first stage; S2, The second stage*.

### Healthcare Safety and Quality

**Figure 2:**
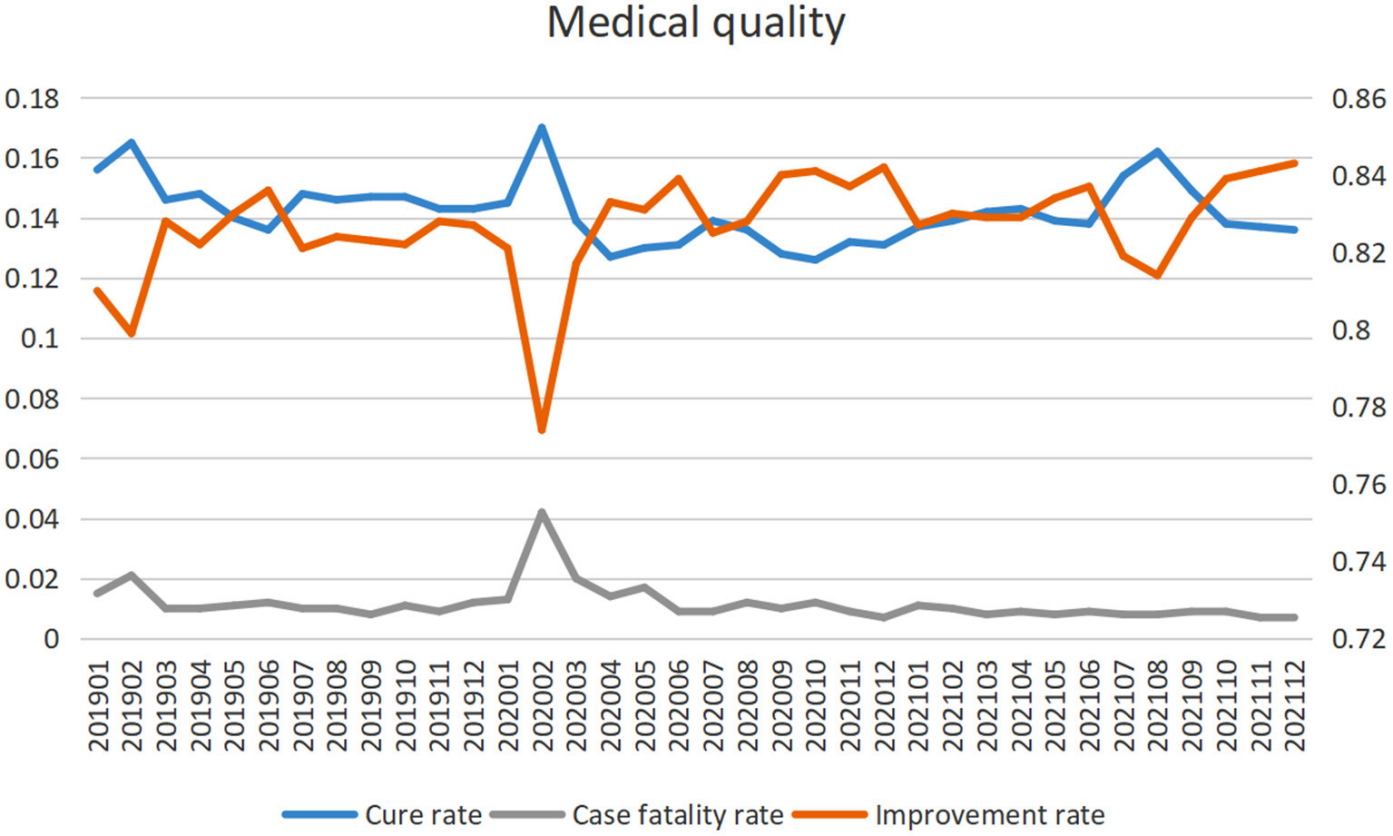
Medical quality trends.

Although the outbreak of the epidemic has affected the medical service capacity, it has not affected the medical safety and quality of hospitals. In this study, the improvement rate of the hospital was significantly increased, the monthly change percentage was 0.1% ((95% CI: 0, 0.1; *P* = 0.013), and the fatality rate was significantly decreased, and the monthly change percentage was −1.6 (−2.6, −0.6; *P* = 0.004). The cure rate did not change significantly (*P* = 0.118) (Table 2; Figure 2).

**Table 2:** Trend analysis of medical service capability.

**Characteristics**	**MPC**	**95% CI**	***P***
Improvement rate	0.1	(0, 0.1)	0.013
Case fatality rate	−1.6	(−2.6, −0.6)	0.004
Cure rate	−0.2	(−0.4, 0)	0.118

## Discussion

A number of professional organizations have issued empirical guidance for adjusting clinical services during the COVID-19 pandemic. Decisions made by administrators and hospital leaders must be supported by clinical data to provide realistic predictions and better inform the intelligent deployment of personnel and resources. Decisions based on clinical data can avoid overestimating the effectiveness of emergency response measures and jeopardizing emergency service delivery. This study analyzed the medical service capacity and medical safety data of tertiary hospitals before the COVID-19 epidemic, during the pandemic, and after the epidemic. The outbreak did not affect the quality and safety of medical care, but only reduced the capacity of medical services in the short term. However, with the development of Internet medical care, the overall medical service capacity of hospitals has increased significantly.

In the post-holiday period in 2020, when the pandemic was at its peak, many cities were under strict lockdowns, and overall health care spending and frequency of use dropped sharply, according to a multicenter study in China. High-risk cities, closed-down cities, and western cities saw a larger decline, and the epidemic led to a significant drop in healthcare utilization during the Spring Festival and post-holiday periods in 2020 compared to 2019, especially in high-risk cities (6). Similar situations have occurred in other countries with the COVID-19 pandemic, such as the United Kingdom, the United States, France, Spain, and Italy (18, 19). Organ transplant operations have decreased significantly, and emergency room visits have decreased dramatically (18, 19). A study in Hong Kong showed that patients waited about 5 h for medical care in early 2020, compared to only about 1 h in the same period in 2019 (20). Patients may delay seeking medical care for a number of reasons, including fear of nosocomial infections, lockdown restrictions, and more. Preventive services have also been affected due to the suspension of routine immunization campaigns due to COVID-19. Due to service issues, some patients' health may worsen due to delays in treatment or prevention.

Since March 2020, multiple hospitals in the United States have experienced a massive migration to virtual care, resulting in a more than 80% reduction in in-person patient visits. At the height of the epidemic, a New York City report noted that Internet consultations increased from <50 per day to more than 1,000 per day, accounting for more than 70% of hospital outpatient visits (21). The outpatient volume of different specialties in different countries such as Italy, the United States and India reported a migration percentage of 60−95% from offline medical care to online medical care (22–24). Internet medical care in the European Union, the United States, India and other countries focuses on the development of telemedicine and mobile medicine, electronic medical records, and provides telemedicine service guidance and rehabilitation recommendations for patients with cardiovascular, dermatology, and stroke patients (25–27). Under the premise of promoting patient health, develop patient medical data, including various examination results, medical information collection and analysis technology. However, with the repeated fluctuations of the epidemic, what we urgently need to solve is to reduce the crowd gathering without affecting the patients' medical treatment.

During the pandemic, the General Hospital of Tianjin Medical University conducts nucleic acid tests for employees twice a week to ensure early detection and control of nosocomial spread of the epidemic. All staff's items are centrally stored in each locker room, and special personnel conduct regular disinfection. Hospitals have implemented multiple measures to deal with infection control. Including pre-examination and triage, strict control of admission gates, strengthening fever clinics, allocating personnel and facilities, strengthening ward access control management, controlling the flow of people in wards, escorting and guiding patients throughout the process, preventing patients from inadvertently running around, attaching importance to surgical management, and during the epidemic Suspension of non-emergency surgeries during outbreak.

Based on the above measures, Tianjin Medical University General Hospital has established an Internet outpatient service platform according to the national conditions. According to the results of this study, the opening of online outpatient clinics has met the medical needs of the majority of patients during the epidemic, and the number of medical visits has increased significantly month by month. At the same time, the number of hospitalizations, the number of emergency departments and the number of surgeries have also increased significantly. Internet medical care not only facilitates patients to seek medical treatment, but also reduces the burden of medical treatment and the service cost of medical institutions, and increases the medical service capacity.

There were some limitations in this study. First, this study is data from one hospital and is not representative of the national sample. Second, this study is only a trend analysis, and the existing data does not have the ability to conduct a detailed analysis of discarded data points affecting medical services.

## Conclusion

COVID-19 temporarily reduced the medical service capacity. With the normalization of epidemic prevention and control, the main business indicators of Tianjin tertiary hospitals have gradually recovered. The operation of Internet medical care during the epidemic has changed the management and operation mode of hospitals to a certain extent, reduced the burden of medical treatment and the service cost of medical institutions, and increased the medical service capacity.

## Data Availability Statement

The raw data supporting the conclusions of this article will be made available by the authors, without undue reservation.

## Author Contributions

JC, LJ, and XS were involved in conception and design and data interpretation for this article. LJ, XZ, DG, JW, MD, and MC were involved in data collection, case diagnosis, and confirmation for this article. JC and LJ were involved in manuscript drafting. JW was involved in data analysis for this article. YS, HZ, and MG were involved critical review in for this article. All authors agree to be accountable for all aspects of the work. All authors contributed to the article and approved the submitted version.

## Funding

This work was supported by the general project of the Science and Technology Project of Tianjin Municipal Health Commission (TJWJ2021MS001), Tianjin Key Research and Development Plan, Key Project of Science and Technology Support (20YFZCSY00010), and Tianjin 131 Innovative Talents Team Training Project in 2016.

## Conflict of Interest

The authors declare that the research was conducted in the absence of any commercial or financial relationships that could be construed as a potential conflict of interest.

## Publisher's Note

All claims expressed in this article are solely those of the authors and do not necessarily represent those of their affiliated organizations, or those of the publisher, the editors and the reviewers. Any product that may be evaluated in this article, or claim that may be made by its manufacturer, is not guaranteed or endorsed by the publisher.
